# *Leishmania* Exosomes/Extracellular Vesicles Containing GP63 Are Essential for Enhance Cutaneous Leishmaniasis Development Upon Co-Inoculation of *Leishmania amazonensis* and Its Exosomes

**DOI:** 10.3389/fcimb.2021.709258

**Published:** 2022-02-03

**Authors:** Alonso da Silva Lira Filho, Emanuella Francisco Fajardo, Kwang Poo Chang, Pauline Clément, Martin Olivier

**Affiliations:** ^1^ Department of Microbiology and Immunology, McGill University, Montréal, QC, Canada; ^2^ Infectious Diseases and Immunity in Global Health Program, The Research Institute of the McGill University Health Centre, Montréal, QC, Canada; ^3^ Department of Microbiology/Immunology, Chicago Medical School, Rosalind Franklin University of Medicine and Science, North Chicago, IL, United States

**Keywords:** *Leishmania*, cutaneous Leishmaniasis, GP63, extracellular vesicles, exosomes GP63-enriched Leishmania exosomes and Leishmaniasis

## Abstract

Protozoan parasites of the genus Leishmania are transmitted by the bite of infected sand flies leading to a wide range of diseases called leishmaniasis. Recently, we demonstrated that *Leishmania* spp.*-*derived exosomes/extracellular vesicles (EVs/LeishEXO) were released in the lumen of the sand fly midgut and to be co-egested with the parasite during the blood meal and that LeishEXO were found to stimulate an inflammatory response conducting to an exacerbated cutaneous leishmaniasis, also it was shown that these vesicles cargo important virulence factors like GP63. Thus, this study aimed to confirm through morphological and proteomic analysis a novel model specificity utilizing another set of GP63-altered *Leishmania amazonensis* parasite strains. Consequently, we proposed to further study the impact of different GP63 vesicle expression levels on their ability to modulate innate inflammatory cell responses, and finally to determine the importance of GP63 vesicle content on the exacerbation of the cutaneous *Leishmania* spp. pathology after their host co-inoculation. Our results revealed that the protein composition of extracted extracellular vesicles were similar to each other and that GP63 was the sole virulence factor changed in the exosomes composition confirming the specificity of the chosen novel model. We further demonstrated that vesicles with different GP63 EVs cargo displayed distinctive macrophage immunomodulatory capabilities at both gene and protein expression *in vitro*. Finally, we showed their diverse impact on the *Leishmania* spp. cutaneous pathology in an *in vivo* setting and confirmed GP63 as a primordial component of the ability of these EVs in augmenting the inflammatory cutaneous response in *Leishmania* spp. infection. Our findings provide new insight on the immune response happening in cutaneous leishmaniasis, shade light on the mechanism behind the host-pathogen interaction occurring in the initial moments of infection, thus creating the opportunity of using them as the target of new pharmacological treatments and vaccinations.

## Introduction

*Leishmania* spp. is a trypanosomatid protozoan parasite transmitted by the bite of infected female phlebotomine sandflies that can lead to a variety of different diseases called Leishmaniasis. From a self-healing cutaneous lesion (Cutaneous Leishmaniasis) to its possibly lethal systemic visceral form (Visceral Leishmaniasis), it is considered by the World Health Organization a neglected vector-borne tropical infection.^1^ It is known that *Leishmania* spp. has an intricate way to overcome the host immune system and to use it to further propagate itself in the host macrophages by using different virulence factors like GP63 (Isnard et al., 2012), a zinc-metalloprotease which can activate important macrophage phosphatases leading to the dephosphorylation of main inflammatory proteins, like the MAP kinases, all converging to the shutdown of the host pro-inflammatory cell response (Gomez et al., 2009).

Exosomes are endosomal vesicles of 30-150 nm of diameter produced by the majority of eukaryotic and prokaryotic cells (Johnstone et al., 1987). These extracellular vesicles have been described to have a role in many physio/pathological contexts like cell-cell communication (Johnstone et al., 1987), immune (Turpin et al., 2015), kidney (Fernández-Llama et al., 2010), cancer (Skog et al., 2008), and infectious diseases (Hosseini et al., 2013). In fact, exosomes are also produced by *Leishmania* spp. and are considered part of its secretome containing the previously mentioned GP63, but also lipophosphoglycan (LPG), and elongation factor 1 (EF-1) (Hassani et al., 2011; Olivier et al., 2012; World Health Organization, 2014).

Furthermore, previous studies demonstrated not only these vesicle`s production but as well their release in the midgut of the sandfly vector and their consequent co-inoculation to the infected host during the insect bloodmeal, worsen the pathology of the cutaneous lesion with increased expression of inflammatory cytokines (Atayde et al., 2015). Nevertheless, questions remained to be answered about the importance of the vesicle content of specific virulence factors on their ability to lead to this exacerbated cutaneous pathology.

Formerly, to better understand a GP63-dependent immune modulation of the macrophage by *Leishmania* spp.-derived exosomes, a comparison of the immunomodulatory properties of vesicles coming from *Leishmania major* (WT) and *L. major* GP63^−/−^ (KO) *in vitro* and *in vivo* was done (Hassani et al., 2014). For this, mass spectrometry studies were performed to check the protein content of both populations of exosomes that showed in the absence of GP63 the distribution of key important proteins greatly differed from its wild type counterpart, including some other known parasite virulence factors. Whereas this study could address certain of its proposed questions, it potentially fell short on specifically addressing the role of GP63 on this immune modulation, because other crucial proteins also had their exosome composition changed by the used knockout strategy. Thus, a study that utilizes a methodology that is capable of precisely answering this important question turned to be essential.

To address this need, our study aimed to confirm a novel model specificity by comparing the morphology and protein content of vesicles derived from transgenic GP63-altered *Leishmania amazonensis*. Consequently, we proposed to further study the impact of different GP63 vesicle expression levels on their ability to modulate innate inflammatory cell responses, and finally to determine the importance of GP63 vesicle content on the exacerbation of the cutaneous *Leishmania* spp. pathology after their host co-inoculation.

## Materials and Methods

### *Leishmania amazonensis* and Cell Culture

A set of transgenic parasites of the species *L. amazonensis* (LV78, MPRO/BR/72/M1845) of virulent clone 12-1 that were transfected with the P6.5 vector alone (control, *L. amazonensis* GP63^WT^), or with the GP63 gene either in the correct (P6.5/1.9, sense mutant, *L. amazonensis* GP63^High^) or in the reverse orientation (P6.5/1.9R, antisense mutant, *L. amazonensis* GP63^Low^), were generated by Dr. Kwang-Poo Chang laboratory. Parasites were grown in SDM culture medium (Schneider’s Drosophila Medium) supplemented with 10% FBS (heat-inactivated fetal bovine serum) and cultured as described (Chen et al., 2000). In summary, transfectants were grown under optimal selective pressure of tunicamycin (TM, Sigma) at 20 µg/ml and before experiments, parasites were cultured without TM until reaching their late stationary phase, then centrifuged at 300 x g (RCF), washed with PBS and used for *in vitro* and *in vivo* experiments accordingly.

Immortalized bone marrow-derived macrophages (B10R) were grown in Dulbecco’s MEM (DMEM) and supplemented with 10% fetal bovine serum (FBS), 5% L-glutamine, and 5% penicillin-streptomycin. Cells were maintained at 37-celsius degrees in a humidified 5% CO2 atmosphere and transferred biweekly. By using a 6-well plate, cells were transferred and used for *in vitro* stimulations which consisted of 5µg of Leishmania-derived exosomes/extracellular vesicles (LeishEXO/EVs) for 4 hours. After this, cells were harvested and prepared according to the protocols necessary for the following techniques: Western blot or qRT-PCR. Supernatant was kept for protein quantification by multiarray ELISA.

### Extraction of *L. amazonensis*-Derived Exosomes/Extracellular Vesicles

To extract exosomes/extracellular vesicles (LeishEXO/EVs) of *L. amazonensis* GP63^WT^, GP63^High^, and GP63^Low^, 800 mL of parasites in late log phase of each strain were centrifuged at 300 x g (RCF) and pelleted. Parasites were then washed 3 times with PBS and centrifuged at 300 x g (RCF) after each washing. Samples were resuspended in RPMI 1640 medium without phenol-red (Life Technologies) then incubated at a shaking incubator at 37°C for 4 hours. After this, the parasite culture was centrifuged at 300 x g (RCF) for 5 minutes to pellet parasites and separate cells from the vesicle-enriched supernatant, which was then collected and centrifuged at 2000 x g (RCF) for 10 minutes. Further, the collected supernatant was filtered through a 0.45-micron syringe filter followed by a 0.20-micron syringe filter. Filtered samples were then centrifuged for 1 hour at 100.000 x g (RCF) at 4°C using a Beckman Coulter Life Sciences, Indianapolis, IN, USA ultracentrifuge (model Optima XPN-90™) and a swing rotor (model SW32.Ti) using open-top thin wall polypropylene tubes (16 x 102 mm), (Beckman Coulter™, Brea, CA, USA). Supernatant was discarded and pellets were collected, pooled together and resuspended in exosome buffer (137 mM NaCl, 20 mM Hepes pH 7.5). Finally, samples were centrifuged for 1 hour at 100.000 x g (RCF) at 4°C, the final pellet was resuspended in exosome buffer in approximately 200uL and stored at -80°C. Protein concentrations of the extracted samples were assessed by microBCA Protein Assay kit according to manufacturer`s manual. (Thermo Scientific - catalog number: 23235).

### Nanoparticle Tracking Analysis (NTA)

*L. amazonensis* GP63^WT^, GP63^High^, and GP63^Low^ EVs preparations were analysed by NTA using a LM-10 Nanosight machine in the laboratory of Dr. Janus Rak at the Research Institute of the McGill University Health Centre. For the determination of particle size and concentration, 3 sequential 30-s videos were acquired of each triplicate using the default settings of the instrument. Exosome buffer was used as the negative control.

### Transmission Electron Microscopic (TEM)

For negative staining, vesicles preparations were coated directly on Formvar/Carbon grids, fixed with 1% glutaraldehyde in 0.1 M sodium cacodylate buffer for 1 min and stained with 1% uranyl acetate for 1 min. Formvar grids covered with isolated vesicles were visualized in the FEI Tecnai 12 120 kV transmission electron microscope. Images were taken with the AMT XR-80C CCD Camera System (Facility for Electron Microscopy Research, McGill University).

### Animals/Ethic Compliance

Male Balb/c mice were infected in their footpad with stationary *L. amazonensis* GP63^WT^ only (5x10^6^/footpad) or co-inoculated with either exosomes/EVs (10 µg/mice) derived from *L. amazonensis* GP63^WT^ (LeishEXO GP63^WT^), GP63^High^ (LeishEXO GP63^High^), or GP63^Low^ (LeishEXO GP63^Low^), having their footpad thickness measured weekly. In another set of experiments, animals were infected with stationary *L. amazonensis* GP63^Low^ alone or with co-inoculated with the LeishEXO (10µg/mice) derived from the 3 distinct parasite strains, also having their footpad thickness measured weekly. The animal study was reviewed and approved by the Facility Animal Care Committee of the Research Institute of the McGill University Hospital Centre (RI-MUHC).

### Quantitative Real-Time – Polymerase Chain Reaction (qRT-PCR)

Total RNA extraction was performed using the TRIzol reagent, following the manufacturer’s protocol. In summary, the samples were treated, to remove genomic DNA fragments with the kit (Promega™). Reverse transcription of RNA into cDNA was performed using Superscript III reverse transcriptase (Invitrogen™) and random hexamers (Invitrogen™), according to the manufacturer’s protocol. cDNA (assayed by NanoDrop™) was added to supermix SYBR Green (Bio-Rad) as well as some gene-specific primers sought in a 96-well plate. The following PCR procedure was then performed in the Touch Real-Time PCR detection system (Bio-Rad™), following the manufacturer’s protocol. The cycles being: 1. 95.0 o C for 3:00 2. 95.0 o C for 0:15 3. TM o C for 0:15 4. 72.0 o C for 0:30 5. Go to 2, 39 times more 6. Melt Curve 65.0 at 95.0°C, increase of 0.5°C by 0:05 seconds the results were analyzed using the ΔΔ method using the group control values for relative comparison. Data were submitted to GraphPad Prism 8™ for statistical analysis using ANOVA by Kruskal-Wallis`s test and uncorrected Dunn`s test for multiple comparisons.

### Western Blot

B10R macrophage proteins were extracted with a lysis buffer based on NET, glycerol, and Igepal. They were then inhibited with a cocktail based on NaF and Na3VO4. Proteins were assayed by Bradford and 10 was suspended in SDS buffer containing bromophenol blue and beta-mercaptoethanol. Protein electrophoresis was then performed on a 10% acrylamide gel with sodium dodecyl sulfate (SDS-PAGE). Proteins were then transferred to a polyvinylidene fluoride (PVDF) membrane using a turbo trans-blot (Bio-Rad™), as indicated by the manufacturer. Samples were then blocked with 5% fat-free milk solution in TBS for 1 hour at RT. Then washed three times with 1X TBST (Tris-buffered saline with Tween 20). Primary antibodies used for the blotting procedure were Leishmania GP63 (R. W. McMaster, University of British Columbia, Vancouver, Canada), Leishmania HSP70 (J. M. Requena, CSIC-UAM, Spain) and Leishmania Arginase (B. Ullman, OHSU – Oregon Health and Science University, USA) which were incubated overnight (16 hours in total). After washing three times, proteins were detected with a secondary anti-mouse antibody conjugated with horseradish peroxidase (HRP) (Amersham™) which had been incubated for 1 hour and washed for 3 times with 1X TBST before been exposed with Prime blot solution and the membranes revealed using a protein radiography machine.

### Multiplex Cytokine Array

The multiplex assay was performed on supernatant of *in vitro* stimulated cells by Eve Technologies using the Bio-Plex 200 system and the Milliplex Mouse Cytokine/Chemokine Magnetic Bead Panel Kit according to their protocol. Values were submitted to GraphPad Prism 8™ for statistical analysis using ordinary one-way ANOVA and uncorrected Fisher`s LSD test for multiple comparisons.

### LC-MS/MS Proteomic Analysis

Proteins of GP63-altered *Leishmania-*derived exosomes/EVs were precipitated with 15% trichloroacetic acid (TCA)/acetone and digested with trypsin at a final concentration of 2 ng/ml. Liquid chromatography-tandem mass spectrometry (LC-MS/MS) was performed at the Institute de Recherches Cliniques de Montreal (IRCM, University of Montreal). Data were searched against the NCBI database of *Leishmania mexicana*, as it has more proteins described and it shares a similar taxonomy of *Leishmania amazonensis*. Before LC-MS/MS, protein digests were re-solubilized under agitation for 15 min in 10 ml of 0.2% formic acid. Desalting/cleanup of the digests was performed by using C18 ZipTip pipette tips (Millipore, Billerica, MA). Eluates were dried down in a vacuum centrifuge and then re-solubilized under agitation for 15 min in 10 mL of 2% ACN/1% formic acid. The column was installed on the Easy-LLC II system (Proxeon Biosystems, Odense, Denmark) and coupled to the LTQ Orbitrap Velos (ThermoFisher Scientific, Bremen, Germany) equipped with a Proxeon nanoelectrospray ion source.

### Bioinformatic Analysis

Protein database searching was performed with Mascot 2.2 Matrix Science) against NCBI and UNIPROT *Leishmania mexicana* protein databases. The mass tolerances for precursor and fragment ions were set to 10 ppm and 0.6 Da, respectively. Trypsin was used as the enzyme allowing for up to 2 missed cleavages. Triplicates of separately analyzed sets of MS/MS data were used for calculation of the Exponentially modified protein abundance index (emPAI) values using Scaffold™ Software. Mascot output files were analyzed by Scaffold™ Software and hits with a minimum of 2 peptides and peptide threshold of 80% and protein identity of 95% were chosen as true hits for further analyses. Gene Ontology (GO) annotations of identified proteins were extracted using Panther database (https://www.panther-db.org) Protein-protein interaction networks of the identified proteins were created using STRING database (https://www.string-db.org) using highest confidence of interaction (>0.900), showing only connected proteins into the network and using a MCL inflation parameter of 3.

### Statistical Analysis

Statistical analyses were performed using uncorrected ANOVA for multiple comparisons on proteomic data, while One-way ANOVA by Kruskal-Wallis test and multiple comparisons by Uncorrected Dunn`s test or 2-way ANOVA with multiple comparisons by uncorrected Fisher LSD`s test for the rest of data acquired. Bars = Mean, Error bars = SEM *p ≤ 0.05, **p ≤ 0.01, ***p ≤ 0.001 and ****p ≤ 0.0001. The data were analyzed using GraphPad Prism software.

## Results

### GP63-Altered *Leishmania amazonensis-*Derived EVs Have Similar Protein Content

In order to specifically assess the importance of GP63 on the immune modulation of the cutaneous leishmaniasis, a set of transgenic parasites of the species *L. amazonensis* (LV78, MPRO/BR/72/M1845) had their endogenous GP63 levels up-or down-regulated by episomal expression of sense and antisense GP63 RNA was chosen for this study (Chen et al., 2000). This way, we could identify the role of this virulence factor on this cutaneous inflammatory response.

**Figure 1:**
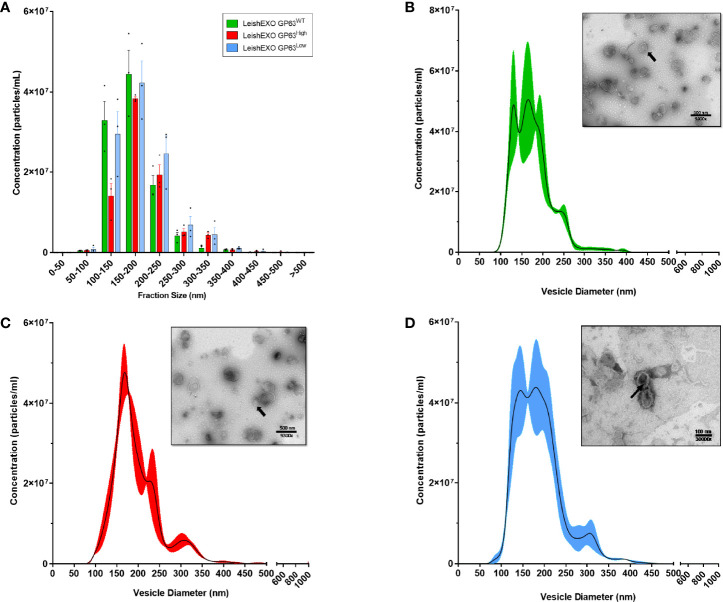
The 3 different populations of *Leishmania amazonensis-derived* EVs showed similar morphologic characteristics when analyzed by Transmission Electronic Microscopy (TEM) and Nanoparticles Tracking Analysis (NTA). **(A)** Grouped distribution of diameter size of various *Leishmania amazonensis*-derived EVs by NTA; **(B–D)** Curve lines of the diameter size distribution of *Leishmania amazonensis* GP63^WT^, GP63^High,^ and GP63^Low^-derived EVs with their correspondent TEM image respectively. Magnification = 9500x or 30000x; Bar = 500nm or 100nm; Arrows = Single EV.

To validate the use of these vesicles as suitable for this methodology and to investigate morphological differences between the extracted EVs, we performed TEM and NTA to check and compare their form and diameter size. Even though coalescence was observed on all strains, vesicles showed exosome-typical patterns with particle populations consisting of double-membraned cup-shaped vesicles with diameters ranging from 50-150nm when observed by TEM, and with similar curve lines of distribution of diameter sizes with peaks on the range of 100-200nm when analyzed by NTA (**Figure 1**), (**Data Sheet S1**). Thus, validating the extracted vesicles as suitable EVs for this study, showing their exosome characteristic morphology and confirming their similarity.

**Figure 2:**
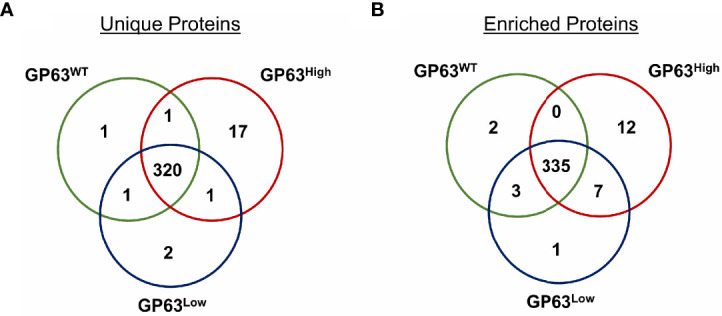
Proteomic analysis of *Leishmania amazonensis* GP63^WT^, GP63^High,^ and GP63^Low^-derived EVs by Mass Spectrometry showed unique and enriched proteins. **(A)** Venn diagram showing the number of shared proteins between LeishEXO GP63^WT^, LeishEXO GP63^High,^ and LeishEXO GP63^Low^ as well as the number of unique proteins of each of the 3 vesicle populations. LeishEXO GP63^High^ had the highest number of detected unique proteins; **(B)** Venn diagram showing the number of shared proteins found to be enriched in each of the 3 vesicle populations as well as the number of proteins with stable levels of detection between the 3 groups. LeishEXO GP63^High^ was found to have the highest number of enriched proteins. ANOVA analysis without corrections with the significance level as p<0,05; Mass spectrometry data analysis performed using Scaffold 4 ™.

To check the protein content of derived vesicles and validate the specificity of GP63 change on these vesicles, we next performed mass spectrometry analysis on the derived exosomes. Seventeen (**Figures 2A, B**) proteins were identified to be part only in the group LeishEXO GP63^High^ having as main common characteristic to be part of various metabolic pathways of the parasite. Additionally, the same characteristic was observed on the proteins characterized as unique on the vesicles LeishEXO GP63^WT^ and GP63^Low^, each group having 1 and 2 proteins classified in such way, respectively (**Table 1**). When analyzing the enrichment of common proteins between the 3 groups, 12 were statistically significant according to uncorrected p-values enriched in the group LeishEXO GP63^High^, not surprisingly GP63 was part of this list, and a protein named Chain A, Crystal Structure of *Leishmania Mexicana* Arginase. Further, 2 proteins were similarly significantly enriched on vesicles GP63WT and 1 protein on LeishEXO GP63^Low^ (**Table 2**). We thus know that the global protein content of each group of vesicles did not greatly differ from their wild-type counterpart and that important known virulence factors were not part of the proteins classified as unique or enriched on both groups. More details of this analysis can be found as supplemental data (**Data Sheet S2**).

**Table 1:** Unique proteins of *Leishmania amazonensis* GP63^WT^, GP63^High,^ and GP63^Low^-derived EVs detected by Mass Spectrometry.

Unique Proteins		
LeishEXO GP63^WT^		
*Protein Name*	*Accession #*	*M.W.(kDa)*
Putative threonine synthase	gi|322489635	74
**LeishEXO GP63^High^**		
*Protein Name*	*Accession #*	
Amp deaminase-like protein	gi|322489528	183
D-lactate dehydrogenase-like protein	gi|322493021	53
Flavoprotein subunit-like protein	gi|322488452	71
Isopentenyl-diphosphate delta-isomerase,putative	gi|322495776	39
Peptidase T, putative, metallo-peptidase, ClanMH,family M20B	gi|322490115	47
Putative calreticulin	gi|322494024	45
Putative fatty acid elongase	gi|322489669	32
Putative fructose-1,6-bisphosphatase, cytosolic	gi|322488101	35
Putative glucosamine-6-phosphate deaminase	gi|322494424	32
Putative glucose-regulated protein 94	gi|322488710	87
Putative mevalonate kinase	gi|322493823	38
Putative prohibitin	gi|322495262	32
Putative ribokinase	gi|322492859	35
Putative serine carboxypeptidase (CBP1)	gi|322490293	52
Putative vacuolar type h+ ATPase subunit	gi|322491875	17
SEC61-like (pretranslocation process) protein,putative	gi|322489303	54
Threonine ammonia-lyase	gi|322488308	37
**LeishEXO GP63^Low^**		
*Protein Name*	Accession #	*M.W.(kDa)*
6-phosphogluconolactonase	gi|322492815	54
Conserved SNF-7-like protein	gi|322495306	26

The different levels of GP63 expression of the parasites led to the detection of unique proteins in each of the 3 derived vesicle populations. LeishEXO GP63^High^ had the highest number of unique proteins, mostly similar to the enzymatic characteristic of GP63, like Peptidase T, putative, metallopeptidase, ClanMH, family M20B. Mass Spectrometry data analysis performed using Scaffold 4 ™.

**Table 2:** Enriched proteins of *Leishmania amazonensis* GP63^WT^, GP63^High,^ and GP63^Low^-derived EVs detected by Mass Spectrometry.

Enriched Proteins		
LeishEXO GP63^WT^		
*Protein Name*	*Accession #*	*M.W.(kDa)*
Putative long-chain fatty Acyl CoA synthetase	gi|322487913	74
Trypanothione reductase	gi|322488146	53
**LeishEXO GP63^High^**		
*Protein Name*	*Accession #*	
Alkyl dihydroxyacetone phosphate synthase	gi|322493404	69
Chain A, Crystal Structure of *Leishmania Mexicana* Arginase In Complex With Inhibitor Abhpe	gi|1018192525	36
Fructose-1,6-bisphosphate aldolase	gi|322490707	41
Glyceraldehyde 3-phosphate dehydrogenase,glycosomal	gi|322493686	39
Glycosomal phosphoenolpyruvate carboxykinase,putative	gi|322492996	58
GP63, leishmanolysin	gi|322489112, gi|322489109	64
Protein disulfide isomerase	gi|322491291	52
Putative 6-phospho-1-fructokinase	gi|322488531	54
Putative glucose-regulated protein 78	gi|322493205	72
Putative hexokinase	gi|322491502	52
Putative pyruvate phosphate dikinase	gi|322489298	101
**LeishEXO GP63^Low^**		
*Protein Name*	Accession #	
Cyclophilin a	gi|322492386	19

LeishEXO GP63^High^ showed the highest number of enriched proteins in comparison to the other groups. Not surprisingly, GP63 was found to be highly expressed in this group, with other proteins of interest like chain A, crystal structure of arginase also enriched on this vesicle population. ANOVA analysis without corrections with the significance level as p<0,05. N=3; Mass spectrometry data analysis performed using Scaffold 4 ™.

We then sought to confirm the mass spectrometry data by performing immunoblotting on both parasites and their derived EVs to verify the level of expression of important detected proteins, like GP63 and Chain A, Crystal Structure of *Leishmania Mexicana* Arginase. (**Figure S1**). In comparison to their respective wild type groups, we observed a higher detection of GP63 on parasites GP63^High^ and their derived EVs, whereas a reduced GP63 detection was observed on parasites GP63^Low^ and their derived EVs, and finally we also observed a higher detection of Arginase on LeishEXO GP63^High^, hence confirming the data coming from the mass spectrometry analysis and the abundance of both GP63 and Chain A, Crystal Structure of *Leishmania Mexicana* Arginase proteins on the analyzed parasites and derived vesicles.

**Figure 3:**
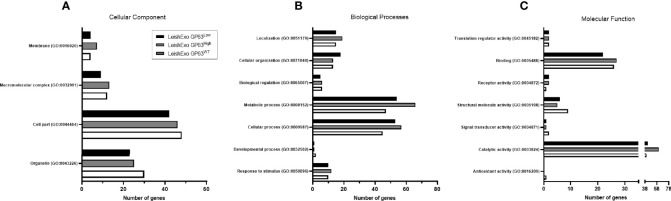
Gene Ontology analysis of various Leishmania amazonensis-derived EV proteins detected by Mass Spectrometry. GO annotations of detected proteins on *Leishmania amazonensis* GP63^WT^, GP63^High,^ *and* GP63^Low^-derived EVs. Cellular components **(A)**, biological processes **(B)**, and molecular functions **(C)** are displayed. Proteins detected on LeishEXO GP63^High^ and GP63^Low^ had more genes classified as related to metabolic processes and cellular processes in comparison to LeishEXO GP63^WT^. Additionally, an increased number of genes classified to the catalytic activity is observed on the group LeishEXO GP63^High^, which correlates with the number of unique proteins in this group related to this molecular function. Analysis performed with Panther classification system at http//:pantherdb.org.

In pursuance of further checking for differences in the protein content of the 3 groups of derived exosomes, gene ontology (GO) analysis was done on each group of proteins. We observed differences in this classification between the groups, more specifically, on the cellular component domain, LeishEXO GP63^WT^ showed more genes classified on the term organelle (GO:0043226) in comparison to the groups of vesicles that had their GP63 expression altered (**Figure 3A**). The same pattern was observed on the term cell part (GO:0044464) which also had fewer genes classified on the groups GP63-altered. Next, when classified regarding the biological process they might participate in, we observed on both LeishEXO GP63^High^ and LeishEXO GP63^Low^ an increased number of proteins categorized on the term cellular process (GO:0009987) and metabolic process (GO:0008152) (**Figure 3B**). Finally, we noticed on the group of vesicles GP63^High^ an increased number of genes classified on the catalytic activity term (GO:0003824) in comparison to both GP63^WT^ and GP63^Low^ (**Figure 3C**). We thus detected some differences in the 3 different vesicle protein content regarding their classification by gene ontology analysis (**Data Sheet S3**).

To further verify changes in the global protein content of the derived vesicles, we generated a scatterplot graph utilizing the normalized log10 EmPAI (Exponentially Modified Protein Abundance Index) values of detected proteins of one group as a baseline curve for comparison with the log10 EmPAI values of another group (**Figure S2**, **Data Sheet S4**). We detected a similar spread of detected protein levels when comparing LeishEXO GP63^WT^ versus GP63^High^, LeishEXO GP63^WT^ versus GP63^Low,^ and LeishEXO GP63^High^ versus GP63^Low^, with a remark to the appearance of clusters of unique proteins in all these comparisons. More details of this comparison can be found on (**Data Sheet S4**).

Moreover, with the final aim of checking that other important parasite virulence factors were not significantly altered by the up-or down-regulation of GP63, we examined the level of other known virulence factors like EF-1, enolase, tryparedozin peroxidase, and others on all groups of vesicles and compared them performing statistical analysis on the detected values by ANOVA. **Table 3** summarizes this data showing that the abundance of the selected parasite virulence factors did not have statistically significant differences between the 3 groups of derived vesicles, leaving GP63 as the main sole factor that had its levels significantly according to uncorrected p-values altered between the studied groups, thus demonstrating the specificity of the model used in this study. After the proteomic validation of the chosen model, we were confident that we could continue further and perform *in vitro* and *in vivo* experiments using the extracted vesicles.

**Table 3:** GP63 expression change did not alter the levels of other known *Leishmania* spp. virulence factors on *Leishmania amazonensis-*derived EVs.

			LeishEXO GP63^WT^		LeishEXO GP63^High^		LeishEXO GP63^Low^		
	Accession #	*M.W.(kDa)*	Average	SEM	Average	SEM	Average	SEM	ANOVA p-value (n=3)
Elongation factor 1-alpha	*gi|322490098*	23	15.87	0.77	12.60	0.65	9.45	3.88	**0.35**
Enolase	*gi|322489720*	46	1.79	0.28	2.37	0.38	1.44	0.48	**0.43**
Tryparedoxin peroxidase	*gi|322489864*	22	3.76	0.57	4.07	0.83	3.59	0.66	**0.92**
Heat shock protein 83-1	*gi|322494527*	81	5.66	1.58	3.73	1.06	6.18	2.61	**0.74**
14-3-3 protein-like protein	*gi|322490909*	30	0.38	0.07	0.24	0.00	0.46	0.19	**0.56**
Calpain-like cysteine peptidase	*gi|322491416*	17	0.33	0.11	0.44	0.05	0.51	0.11	**0.59**
Surface antigen-like protein	*gi|322488233*	74	1.4	0.13	1.23	0.06	0.84	0.20	**0.14**
GP63, leishmanolysin	*gi|322489112*	64	1.27	0.03	2.08	0.18	1.23	0.05	**0.0078**

Expression levels of other important virulence factors, like elongation factor 1-alpha, were assessed and showed stable amounts on the 3 vesicle populations. Numbers in EmPAI. ANOVA without corrections with the significance level as p < 0,05.

### GP63-Altered *Leishmania amazonensis-*Derived EVs Elicit Different Inflammatory Responses on Macrophages

**Figure 4:**
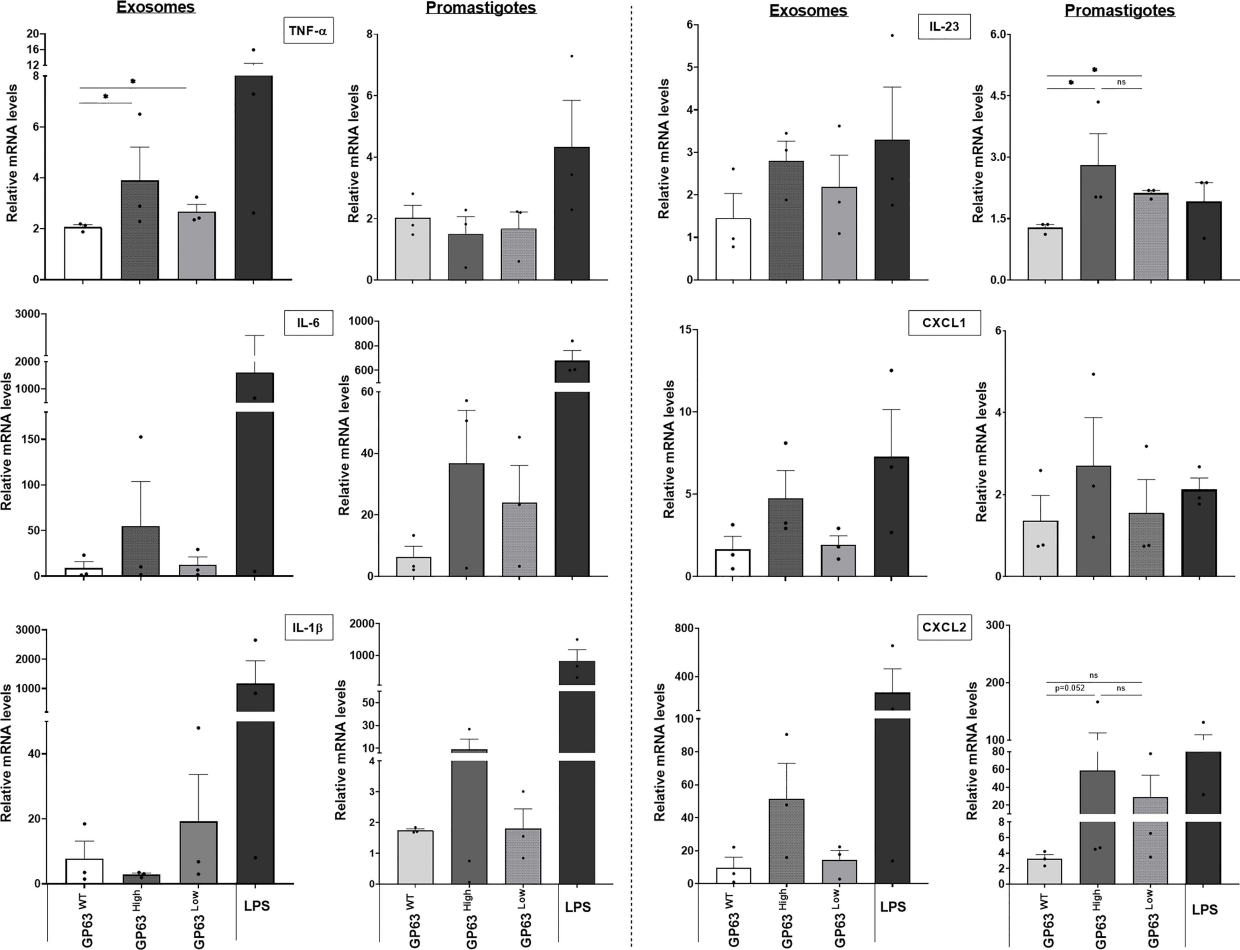
Impact of various *Leishmania amazonensis* species and their derived EVs stimulation on the inflammatory response gene expression of innate immunity cells. Analysis by qrt-pcr of key inflammatory cytokines and chemokines of immortalized bone marrow-derived macrophages (B10Rs), after stimulation for 4 hours with either the 3 different populations of *Leishmania amazonensis*-derived EVs or the 3 strains of *Leishmania amazonensis* live parasites; One-way ANOVA by Kruskal-Wallis test and multiple comparisons by Uncorrected Dunn`s test; *p < 0.05. No symbol, nonsignificant; Bars= Average ± SEM. N=3.

To further study the impact of different GP63 vesicle content on their ability to modulate innate inflammatory cell responses, we performed *in vitro* stimulations of immortalized bone-marrow-derived macrophages with the 3 groups of *L. amazonensis*-derived EVs. Thus, qRT-PCR was performed to check the expression of mRNA of TNF-α, IL-6, IL-1β, IL-23, CXCL1, and CXCL2 by stimulated macrophages. First, we observed that the mRNA expression of TNF-α had its level significantly according to uncorrected p-values modulated depending on the GP63 content of the vesicles used for the stimulation, displaying higher levels of expression in the group that was stimulated with GP63^High^ vesicles in comparison to levels found on cells stimulated with LeishEXO GP63^WT^. In a similar way, but to a smaller degree, we observed increased TNF-α mRNA levels in the group stimulated with LeishEXO GP63^Low^ in contrast to the ones found in the group stimulated with LeishEXO GP63^WT^. Furthermore, we detected this same trend of increased gene expression when checking on IL-6, IL-23, CXCL1, and CXCL2 mRNA levels, but ultimately these were not considered significant following statistical analysis (**Figure 4**).

Additionally, to check how different this EVs immunomodulation is from the one caused by their parasite counterpart, we performed assays using whole promastigotes from which these vesicles are derived from. We noticed that not all cytokines and chemokines mRNA expression followed the same pattern of gene expression caused by the LeishEXO stimulations on macrophages, with the example of TNF-α which independently of the parasite GP63 content, displayed similar mRNA levels of this cytokine between the stimulated cells, and IL-1β which showed a different pattern of gene expression of this cytokine to the levels found on cells stimulated with their derived vesicles (**Figure 4**). Ultimately, we could observe that the GP63 content of the vesicles leads to significant according to uncorrected p-values differential modulation of the macrophage gene expression of TNF-α, and the gene expression followed by this stimulation not always follows the same pattern encountered on macrophages stimulated with their parasite counterpart.

**Figure 5:**
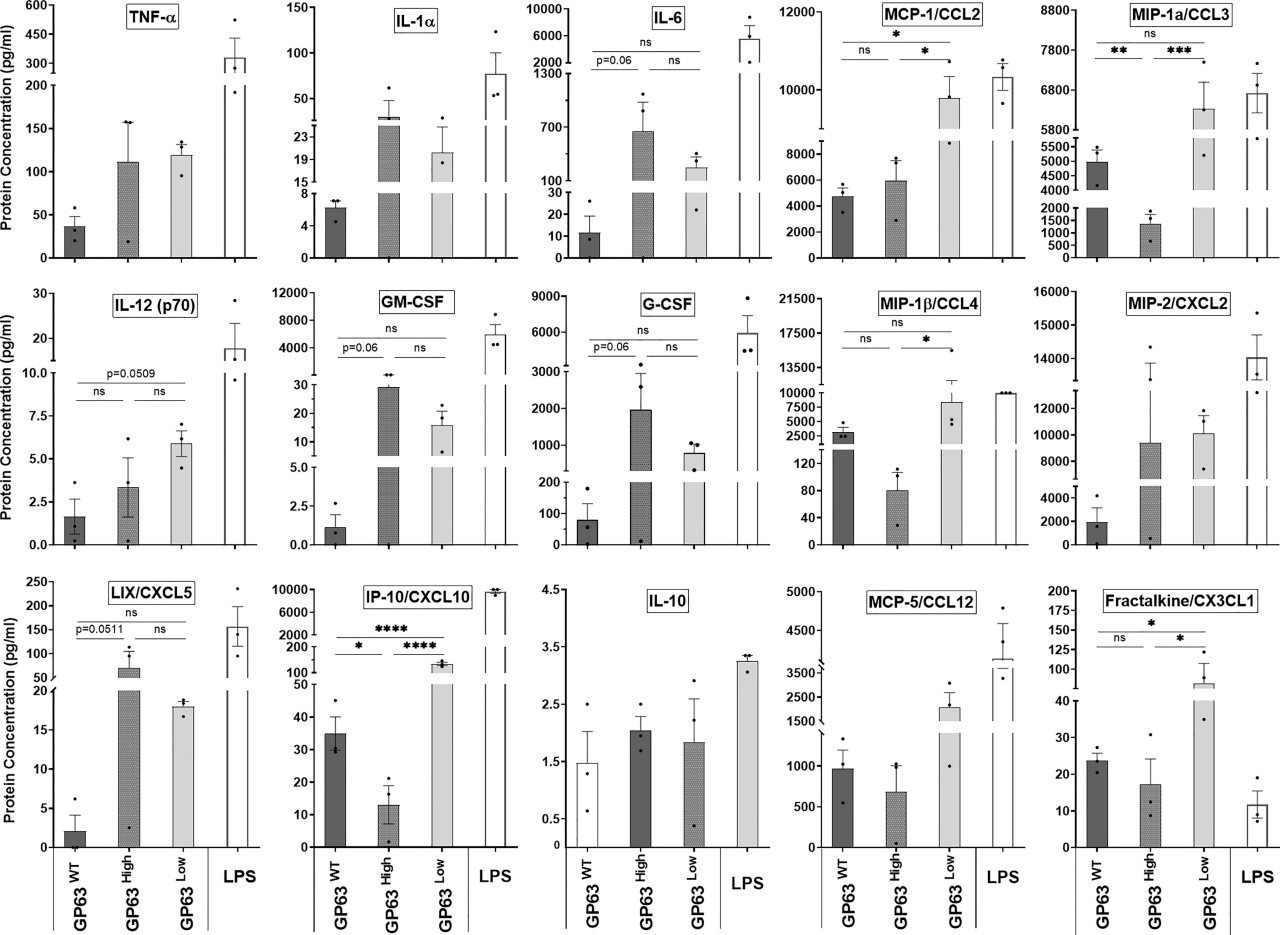
Impact of various *Leishmania amazonensis* species and their derived EVs stimulation on the inflammatory response protein expression of innate immunity cells. Supernatant protein levels of immortalized bone marrow macrophages (B10Rs) *in-vitro* stimulated with *Leishmania*-derived EVs measured by multiarray ELISA assay; Ordinary one-way ANOVA test with multiple comparisons by Uncorrected Fisher`s LSD test; *p < 0.05; **p < 0.01; ***p < 0.001; ****p < 0.0001; No symbol, nonsignificant; Bars=Average ± SEM; N=3.

We next tested whether the gene expression of certain inflammatory cytokines and chemokines caused by LeishEXO stimulations on macrophages results in the abundance of proteins produced by these cells. For this, we performed a multiplex cytokine array on the supernatant coming from *in vitro* stimulated macrophages to assess the protein concentration of various cytokines and chemokines in this solution and to check for any difference in protein abundance levels. We noticed a trend in which the protein concentrations of IL-1α, IL-6, GM-CSF, G-CSF, and CXCL5 were the highest in the group stimulated with LeishEXO GP63^High^ in comparison to the levels found in the 2 other groups, but ultimately these differences were not considered significant after statistical analysis (**Figure 5**). In contrast to that, we observed that the protein levels of CCL2, CCL3, IL-12, CCL4, CXCL10, and CX3CL1 were the highest, and deemed significant according to uncorrected p-values, in the supernatant of the cells stimulated with LeishEXO GP63^Low^, especially when compared to the levels found on the group LeishEXO GP63^High^, which inversely displayed the lowest levels of protein concentration of these cytokines and chemokines on these occasions (**Figure 5**). In summary, we detected that the gene expression of certain cytokines and chemokines were followed by the abundance of their proteins by the stimulated cells and that the GP63 content of vesicles leads to a differential response on this protein production by the macrophages. With these findings in hands, we concluded that it would be important to check if the differences found *in vitro* would be similarly found in an *in vivo* model.

### GP63 Vesicle Cargo Is Essential for the EVs-Driven Hyperinflammatory Cutaneous Response on Cutaneous Leishmaniasis

**Figure 6:**
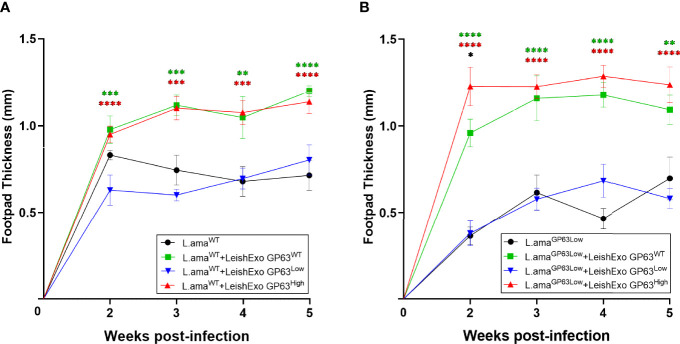
Impact of various *Leishmania amazonensis-*derived EVs on *Leishmania amazonensis* infection. **(A)** *In-vivo* infection model using males Balb/c mice infected on their footpads with *Leishmania amazonensis* wild-type strain co-inoculated with EVs derived from either *Leishmania amazonensis* GP63^WT^, GP63^Low,^ or GP63^High^. ✱ = L.ama^WT^ vs L.ama^WT^+LeishEXO GP63^WT^ **;** ✱ = L.ama^WT^ vs L.ama^WT^+LeishEXO GP63^High^ **;** ✱ = L.ama^WT^ vs L.ama^WT^+LeishEXO GP63^Low^; L.ama^WT^ = *Leishmania amazonensis* GP63^WT^. **(B)** *In-vivo* infection model using males Balb/c mice infected on their footpads with *Leishmania amazonensis* GP63^Low^ strain co-inoculated with EVs derived from either *Leishmania amazonensis* GP63^WT^, GP63^Low^ or GP63^High^; ✱ = L.ama^GP63Low^ vs L.ama^GP63Low^+LeishEXO GP63^WT^ **;** ✱ = L.ama^GP63Low^ vs L.ama^GP63Low^+LeishEXO GP63^High^ **;** ✱ = L.ama^GP63Low^ vs L.ama^GP63Low^+LeishEXO GP63^Low^; ✱ = L.ama^GP63Low^ +LeishEXO GP63^WT^ vs L.ama^GP63Low^ +LeishEXO GP63^High^ L.ama^GP63Low^ = *Leishmania amazonensis* GP63^Low^. 2-way ANOVA with multiple comparisons by uncorrected Fisher LSD`s Test; n=6; *p < 0.05; **p < 0.01; ***p < 0.001; ****p < 0.0001; Error bars = SEM; No symbol, non-significant.

To determine the importance of GP63 vesicle content on the exacerbation of the cutaneous Leishmania spp. pathology after their host co-inoculation, we infected male Balb/c mice on their footpads with *L. amazonensis* GP63^WT^ only or co-inoculated with LeishEXO GP63^WT^, GP63^High^, or GP63^Low^ and followed the infection by checking the mice footpads swelling weekly. After 5 weeks of infection, we observed that mice that received both LeishEXO GP63^WT^ and LeishEXO GP63^High^ displayed significant according to uncorrected p-values increased footpad swelling in comparison to the one found in animals infected with *L. amazonensis* GP63WT only, whereas animals that were co-inoculated with LeishEXO GP63^Low^ showed equivalent footpad swelling to the one found on animals infected with the parasites only (**Figure 6A**).

Finally, we tested whether the observed similarly increased levels of footpad swelling on animals co-inoculated with LeishEXO GP63^WT^ and GP63^High^ was occasioned by the use of *L. amazonensis* GP63^WT^, as baseline infection, which, considering the already present levels of GP63 on these parasites, would lead the group LeishEXO GP63^High^ to reach of a theoretical biological limit for the action of this virulence factor on the increased cutaneous pathology, we infected male Balb/c mice on their footpads with *L. amazonensis* GP63^Low^ only or co-inoculated with LeishEXO GP63^WT^, GP63^High^, or GP63^Low^. At first, after 2 weeks of infection, we detected that the footpad swelling of animals co-inoculated with LeishEXO GP63^High^ was significantly according to uncorrected p-values higher in comparison to the levels found on animals co-inoculated LeishEXO GP63^WT^, but this difference subdued and it was not considered statically significant thereafter and displayed similar footpad swelling to the one displayed on animals co-inoculated with LeishEXO GP63^WT^. Once again, we observed that animals that received vesicles GP63^Low^ displayed similar levels as the ones found on animals infected with *L. amazonensis* GP63^Low^ only (**Figure 6**). In conclusion, we could observe that the GP63 content of *L. amazonensis*-derived vesicles impacted their ability to lead to an increased cutaneous pathology when co-inoculated with the parasite and that differential GP63 levels on vesicles lead to the display of distinct footpad swellings on co-inoculated animals.

## Discussion

*Leishmania* spp. GP63 is the main subject of many studies that identified it as a key virulence factor capable of many diverse effects on the macrophage cell biology and its response to the parasite infection (Brittingham et al., 1995; Yao et al., 2003; Contreras et al., 2010; Olivier et al., 2012). However, an understanding of the importance of this metalloprotease in the composition of *Leishmania* spp.-derived EVs and their immunomodulatory functions is sparse and coming from studies that lacked specificity when addressing these answers. Here, we confirmed the specificity of a novel model that utilizes a set of transgenic GP63-altered *L. amazonensis* and their derived EVs. We further showed that these vesicles displayed different macrophage immunomodulatory capabilities at both gene and protein abundance depending on the level of GP63 EV cargo, and finally demonstrated their diverse impact on the *Leishmania* spp. cutaneous pathology in an *in-vivo* setting and confirmed GP63 as a primordial component of the ability of these EVs in augmenting the inflammatory cutaneous response in *Leishmania* spp. infection.

First, we showed that the 3 groups of vesicles (LeishEXO GP63^WT^, GP63^High,^ and GP63^Low^) are morphologically similar. On further MS studies, we proved the specificity of this model, showing that the global protein content of derived vesicles of the 3 species of *L. amazonensis* did not greatly differ between them. When knockout models were used in previous studies, other virulence factors were altered besides the GP63 (Hassani et al., 2014), which complicates the exact interpretation of which specific role GP63 has in the biological process of immunomodulation of these vesicles. Then, the validation of the transgenic models used here surely improved significantly the specificity of the results found.

Next, we confirmed the different levels of GP63 of parasites were followed by the same distinct abundance of this protein on their derived vesicles, matching the data coming from MS analysis. Most importantly, when utilizing proteomic and bioinformatic analysis of the vesicle’s protein content, we verified that other important *Leishmania* spp. virulence factors, like EF-1 (Nandan et al., 2002), Enolase, and HSP83-1 were not significantly altered by the down- or up-regulation of GP63 on their parasite counterpart. This was also observed when we analyzed the unique and enriched proteins of each group, which did not display any other important virulence factor as part of them. Contrarily, studies that utilized a knockout approach of GP63 on the parasites and their derived vesicles, showed that the levels of main virulence factors changed and that the lack of GP63 modified their protein composition (Hassani et al., 2014). Interestingly, studies that utilized parasites and vesicles HSP100-/- also displayed significant changes in their protein composition in comparison to their wildtype counterpart, suggesting this may be a common trait of knockout models of important parasite virulence factors and its effect on derived EVs protein composition (Hassani et al., 2014). So, the specificity of the models used here an important advance in how future studies may be conducted.

On the other hand, we believe that differences found on Chain A, Crystal Structure of *L. mexicana* Arginase expression may not alter the specificity of this model, since this enzyme is a minor virulence factor of *Leishmania* spp., acting on the parasite protection from reactive oxygen species in the macrophage phagosome on its late amastigote stage, which EVs do not have a direct impact, whereas GP63 acts since the crucial initial moments of the infection, preventively downregulating the cell inflammatory pathways, facilitating the parasite entrance and survival on its promastigote stage into the cell, thus increasing its infectivity to the mammalian host (Brittingham et al., 1999; Joshi et al., 2002; McGwire et al., 2003; Silverman et al., 2010; Badirzadeh et al., 2017).

Additionally, after bioinformatic analysis, small changes in the Gene Ontology classification were observed between the different EV groups but with no notable interest. In fact, none of the observed differences would impact the specificity of the used model since they did not display any significant change on the global protein content of vesicles. Further, scatterplot analysis using the global protein content of the 3 EV groups displayed a very similar spread of proteins, firmly suggesting that the protein composition of these vesicles is similar, contrasting with the report that *L. major* GP63 KO were showing a significantly different EVs enriched protein profiles in comparison to *L. major* wild type (Hassani et al., 2014).

Following the confirmation of the specificity of this novel model, we next tested the impact of different GP63 vesicle cargo on their ability to modulate innate inflammatory cell responses. Previous reports showed that the presence or lack of GP63 on these EVs lead to different gene expression of pro-inflammatory cytokines by macrophages, hinting at the importance of this virulence factor on this crucial immunomodulation (Hassani et al., 2014). However, here we found that different GP63 content of vesicles leads to significant differential modulation of the macrophage gene expression of at least TNF-α and that the gene expression followed by this stimulation not always follows the same pattern encountered on macrophages stimulated with their parasite counterpart. This observation is in accordance with previous reports that have shown the inflammatory capabilities of both *Leishmania* spp.-derived vesicles and their parasite counterpart have different impacts on the macrophage inflammatory response since both have different GP63 repertoire in their composition (Santarem et al., 2013). Next, confirming our thoughts that GP63 is a chief component of *Leishmania-ssp.-*derived EVs capability to modulate innate immune cells responses, we found that this inflammatory gene expression was followed by the protein abundance of important inflammatory cytokines and chemokines by the stimulated cells, and that different GP63 content of vesicles leads to distinctive protein production by macrophages. To our knowledge, this is the first time a study shows these findings using both approaches - gene and protein levels of expression.

Considering the many known direct and indirect anti-inflammatory functions of GP63 on transcription factors like AP-1, NF-κB, STAT-1, and various signaling proteins like IRAK-1, JAK-1, and MAP Kinases (Blanchette et al., 1999; Gregory et al., 2008; Halle et al., 2009; Contreras et al., 2010; Olivier et al., 2012) surprisingly we observed an significant increase of TNF-α gene expression by macrophages stimulated with LeishEXO GP63^High^ in comparison to both vesicles with normal and lower levels of GP63. This comes in contrast to our initial hypothesis that higher amounts of GP63 on these vesicles would lead to the reduction of the production of inflammatory cytokines and chemokines by these cells. Even if not statistically significant, the observed trend of increased gene expression of IL-6, IL-23, CXCL1, and CXCL2 are important and should be taken into consideration when interpreting these findings, which also gave us a hint of the increased inflammatory properties of LeishEXO GP63^High^. In addition to this, the observed trend of increased protein abundance of IL-1α, IL-6, GM-CSF, G-CSF, and CXCL5 in the group stimulated with LeishEXO GP63^High^ and the significant increase of CCL2, CCL3, IL-12, CCL4, CXCL10 and CX3CL1 production by macrophages stimulated with LeishEXO GP63^Low^ suggest that each group of vesicles modulate the macrophage inflammatory response towards divergent adaptive immunity polarizations (Silverman et al., 2010).

Altogether, this data suggests the existence of a fine-tuning of the amount necessary of GP63 to act as an anti-inflammatory protein on mammalian cells, and that when more than the necessary quantities are present, this virulence factor might act in the opposite direction of its initial functions of downregulating the macrophage inflammatory response. Thus, knowing this is a promiscuous factor (Olivier et al., 2012) when increased amounts of GP63 are present and acting in the macrophage cytoplasm and organelles, this would result in a loss of specificity of its protease activity, leading to unspecific cleavage of proteins that eventually would stress the cell. This hypothesis is supported by studies that also suggest this inflammatory activity of GP63 of *Leishmania* spp., displaying its ability to increase the production of TNF-α by macrophages. (Arango Duque et al., 2014). Moreover, these inflammatory properties can also be found on other protozoan organisms like *Trypanosoma cruzi* (Trocoli Torrecilhas et al., 2009), suggesting GP63 indeed may have other than anti-inflammatory roles and that this property is conserved in this class of organisms, but other organisms also displayed such inflammatory characteristic (Alaniz et al., 2007; Bhatnagar and Schorey, 2007; Oliveira et al., 2010; Cheng and Schorey, 2013). Again, this GP63 fine-tune existence is supported by our finding that the response on cells stimulated with LeishEXO GP63^Low^ also showed a trend of increased gene expression of inflammatory cytokines, indicating that low levels of GP63 would lead to disruption of this fine-tune and to permissiveness of an inflammatory response by macrophages caused by LeishEXO per se. This corroborates to further studies with the aim of addressing the inflammatory capabilities of *Leishmania* spp. GP63 in macrophages and the impact of this response on the infectivity of this parasite and the course of infection.

In addition to testing the impact of different GP63 EV cargo on their ability to modulate innate inflammatory cell responses in an *in vitro* setting, we went further and tested whether the observed differences are translated into different inflammatory cutaneous responses *in vivo*. Previous studies have shown that the level of GP63 in *Leishmania* spp. have a big impact on the progression of the cutaneous infection (Thiakaki et al., 2006). It is known that low levels of GP63 in parasites used *in vitro* and *in vivo* models resulted in decreased cutaneous disease in comparison to animals that were infected with wild type parasites, whereas high levels of GP63 in parasites resulted in increased cutaneous response in animals (Thiakaki et al., 2006). In contrast, what is unknown is whether the GP63 EV cargo would also alter the progression of this infection when they are co-inoculated with the parasite in the skin. Here, we show that infected animals co-inoculated with both LeishEXO GP63^WT^ and LeishEXO GP63^High^ displayed similar significant increased footpad swelling, whereas animals that were co-inoculated with LeishEXO GP63^Low^ showed equal footpad swelling to the one found on animals infected with the parasites only, demonstrating that GP63 is primordial to the exosomes ability on exacerbating the cutaneous inflammatory response and that different GP63 EV cargo lead to different cutaneous pathologies. Also, it is important to notice that normal levels of other virulence factors found on LeishEXO GP63^Low^ were not enough to preserve its abilities to increase the cutaneous pathology, showing once again the importance of GP63 EV cargo in this skin hyperinflammatory pathology.

Although interestingly we noticed the same level of footpad swelling on both LeishEXO GP63^WT^ and LeishEXO GP63^High^ groups, this data suggests the existence of a biological celling for the functions of GP63 on its ability to facilitate the parasite infectivity when interacting with the phagocyte. As we have seen on the data coming from the *in vitro* experiments, higher than normal levels of GP63 do not translate into lower levels of inflammation by the macrophage, in fact, the opposite, thus indicating that higher than normal levels of GP63 not necessarily lead to an increased cutaneous pathology *in vivo*, but it is prudent to consider that our findings also suggest that this biological celling of *Leishmania* spp. GP63 is reached on the group LeishEXO GP63^WT^ since we utilized parasites GP63^WT^ as the baseline infection in all groups, effectively making the effects of LeishEXO GP63^High^ not measurable in this trial. Thus, to take into account this possibility, we used *L. amazonensis* GP63^Low^ as a baseline infection in a second set of *in vivo* experiments, and not only we demonstrated that if this biological ceiling is not reached, LeishEXO GP63^High^ is capable of causing an augmented cutaneous response in comparison to the LeishEXO GP63^WT^ group, but we also rescued the ability of parasites GP63^Low^ of causing a cutaneous response comparable to the one caused by parasites with normal levels of this virulence factor. Once more, this demonstrates the importance of GP63 EV content on this response and that the first moments of this host-parasite interaction are crucial in the outcome and progression of the infection caused by *Leishmania* spp. Furthermore, the loss of significance of the difference found between the groups LeishEXO GP63^High^ and GP63^WT^ after the second week indicate that when the parasite enters its amastigote stage, and start its late phase of the infection, the effects of LeishEXO GP63^High^ on the parasite infectivity at this initial interaction subdue due to the existence of mechanisms of survival and proliferation of this particular stage of the parasite that are out of the scope of the early actions of LeishEXO.

In conclusion, we confirmed the specificity of a novel model that utilizes a set of transgenic GP63-altered *L. amazonensis* and their derived EVs. We further demonstrated that vesicles with different GP63 EV cargo displayed distinctive macrophage immunomodulatory capabilities at both gene expression and protein production *in vitro*. Finally, we showed their diverse impact on the *Leishmania* spp. cutaneous pathology in an *in vivo* setting and confirmed GP63 as a primordial component of the ability of these EVs in augmenting the inflammatory cutaneous response in *Leishmania* spp. infection. Our findings provide new insight on the immune response happening in cutaneous leishmaniasis, sheer light on the mechanism behind the host-pathogen interaction occurring in the initial moments of infection, putting GP63 and *Leishmania* spp.*-*derived EVs in the center of this battle, thus creating the opportunity of using them as the target of new pharmacological treatments and vaccinations.

## Data Availability Statement

The original contributions presented in the study are included in the article/**Supplementary Material**, further inquiries can be directed to the corresponding author.

## Ethics Statement

Animal experiments were performed in compliance with the Canadian Council on Animal Care (CCAC) Guidelines, and McGill University Animal Care Committee (UACC). The approved animal use protocol number is 7791.

## Author Contributions

MO obtained grant support. MO and AF discussed and elaborated the study. KC generated and provided the mutated Leishmania. AF, MO, and PC performed experiments and analyzed data. AF and PC did the figures and tables. AF and EF wrote the first draft. MO, KC, EF, and AF revised and wrote the final manuscript. All authors contributed to the article and approved the submitted version.

## Funding

M.O. research is supported by the Canadian Institute of Health Research (CIHR; Grant PJT-159765) and the Natural Sciences and Engineering Research Council of Canada (NSERC; Discovery Grant RGPIN- 2018-03849).

## Conflict of Interest

The authors declare that the research was conducted in the absence of any commercial or financial relationships that could be construed as a potential conflict of interest.

## Publisher’s Note

All claims expressed in this article are solely those of the authors and do not necessarily represent those of their affiliated organizations, or those of the publisher, the editors and the reviewers. Any product that may be evaluated in this article, or claim that may be made by its manufacturer, is not guaranteed or endorsed by the publisher.
