# Supplementary material for: Leishmania Exosomes/Extracellular Vesicles Containing GP63 Are Essential for Enhance Cutaneous Leishmaniasis Development Upon Co-Inoculation of Leishmania amazonensis and Its Exosomes
Source: Front Cell Infect Microbiol. 2022 Feb 3;11:709258. doi: 10.3389/fcimb.2021.709258 (PMC8851419; doi:10.3389/fcimb.2021.709258)
Supplement: Supplementary file 5 [file Image_1.pdf]

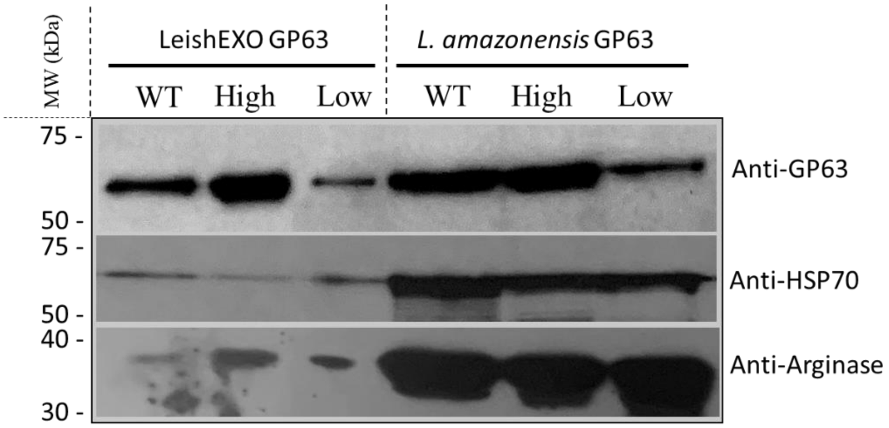

	Accession #	Average GP63WT	SEM	Average GP63high	SEM	Average GP63low	SEM	ANOVA p-value
GP63, leishmanolysin	gi|322489112	33.7	1.0	81.3	5.4	32.7	1.5	0.00022
Heat shock protein 70, partial	gi|281426622	84.7	69.1	54.3	22.3	44.0	35.9	0.88
Chain A, Crystal Structure Of Leishmania Mexicana Arginase In Complex With Inhibitor Abhpe	gi|1018192525	3.0	0.5	24.7	5.5	5.0	1.9	0.018

Figure 1 Supplemental. Verification of proteomic data.

(A) Analysis of protein content of the 3 different strains of *Leishmania amazonensis* and their derived exosomes by western blot confirmed the different levels of GP63 expression detected by MS analysis. Enriched protein levels of chain A, crystal structure of arginase on LeishEXO GP63^{High} were also confirmed. HSP70 was used as loading control but its expression is variable between the different groups; (B) Tabular description of detection levels of GP63, HSP70, and Arginase by MS analysis. Numbers in peptide counts. ANOVA analysis without corrections with the significance level as p<0,05. N=3.
