# Supplementary material for: Leishmania Exosomes/Extracellular Vesicles Containing GP63 Are Essential for Enhance Cutaneous Leishmaniasis Development Upon Co-Inoculation of Leishmania amazonensis and Its Exosomes
Source: Front Cell Infect Microbiol. 2022 Feb 3;11:709258. doi: 10.3389/fcimb.2021.709258 (PMC8851419; doi:10.3389/fcimb.2021.709258)
Supplement: Supplementary file 6 [file Image_2.pdf]

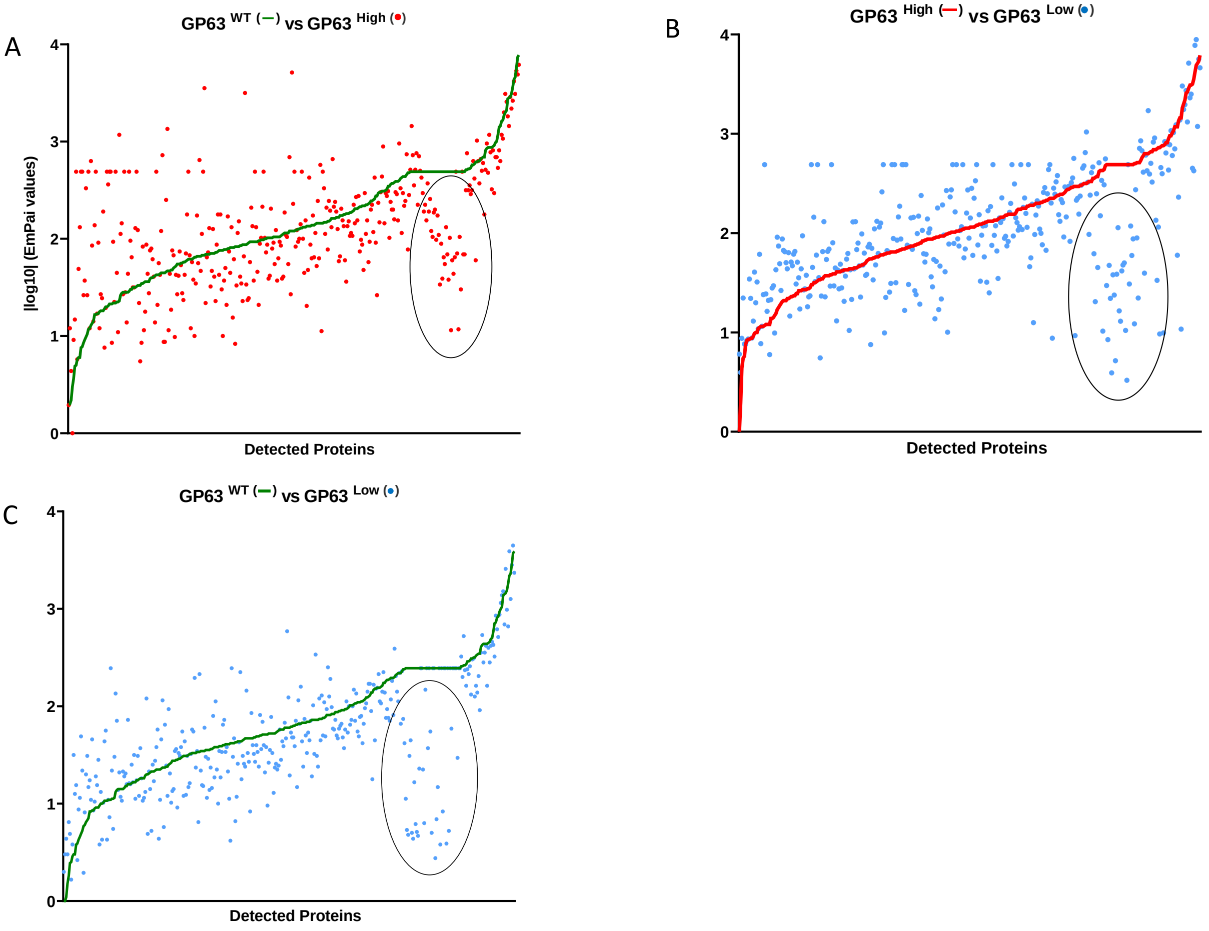


**Figure 2 Supplemental. Comparative proteomic analysis of detected proteins of various *Leishmania amazonensis*-derived exosomes.**

Comparative curve lines: (A) LeishExo GP63$^{WT}$ vs GP63$^{High}$; (B) LeishExo GP63$^{WT}$ vs GP63$^{Low}$; (C) LeishExo GP63$^{High}$ vs GP63$^{Low}$. |log10.emPAI| = y+ log10.emPAI, where emPAI is the exponentially modified protein abundance index, defined as emPAI = 10$^{PAI}$-1, where PAI (Protein Abundance Index) denotes the ratio of observed to observable peptides (N$_{observed}$/N$_{observable}$) and y is a constant defined by the minimum log10.emPAI value of both studied groups. Circled proteins = unique proteins of each analyzed group.
